# Identification of solamargine as a cisplatin sensitizer through phenotypical screening in cisplatin-resistant NSCLC organoids

**DOI:** 10.3389/fphar.2022.802168

**Published:** 2022-08-10

**Authors:** Yi Han, Jianquan Shi, Ziwei Xu, Yushan Zhang, Xiaoqing Cao, Jianhua Yu, Jie Li, Shaofa Xu

**Affiliations:** ^1^ Department of Thoracic Surgery, Beijing Chest Hospital, Capital Medical University and Beijing Tuberculosis and Thoracic Tumor Research Institute, Beijing, China; ^2^ Department of Critical Care Medicine, Beijing Chest Hospital, Capital Medical University and Beijing Tuberculosis and Thoracic Tumor Research Institute, Beijing, China; ^3^ Department of Oncology, Wang Jing Hospital of China Academy of Chinese Medical Sciences, Beijing, China; ^4^ Department of Oncology, Beijing Chest Hospital, Capital Medical University and Beijing Tuberculosis and Thoracic Tumor Research Institute, Beijing, China

**Keywords:** lung cancer, patient-derived organoids, cisplatin resistance, solamargine, hedgehog

## Abstract

Although Cisplatin (DDP) is a widely used first-line chemotherapy medication, DDP resistance is one of the main causes of treatment failure in advanced lung cancer. Therefore, it is urgent to identify DDP sensitizers and investigate the underlying molecular mechanisms. Here we utilized DDP-resistant organoids established from tumor biopsies of patients with relapsed lung cancers. In this study, we identified Solamargine as a potential DDP sensitizer through screening a natural product library. Mechanically, Solamargine induced G0/G1-phase arrest and apoptosis in DDP-resistant lung cancer cell lines. Gene expression analysis and KEGG pathway analysis indicated that the hedgehog pathway was suppressed by Solamargine. Moreover, Gli responsive element (GRE) reporter gene assay and BODIPY-cyclopamine binding assay showed that Solamargine inhibited the hedgehog pathway via direct binding to SMO protein. Interestingly, Solamargine and DDP showed a synergetic effect in inhibiting DDP-resistant lung cancer cell lines. Taken together, our work herein revealed Solamargine as a hedgehog pathway inhibitor and DDP-sensitizer, which might provide a new direction for further treatment of advanced DDP-resistant lung cancer patients.

## Introduction

Lung cancer is the leading cause of death worldwide and accounts for around 18.4% of total cancer-associated death in 2018 (Bray et al., 2018; Siegel et al., 2020). Despite the progress in novel targeted treatment regimens, chemotherapy, especiallyCisplatin-based treatment, is still the first-line treatment for relapsed and advanced lung cancer patients. However, the development of Cisplatin resistance is almost inevitable and results in treatment failure (Kaur et al., 2014; Sun et al., 2016). Thus, it is urgent to find biomarkers and targets to predict and overcome Cisplatin resistance.

Patient-derived organoids (PDOs) have been exploited as novel and effective preclinical models in drug response tests and personalized therapy design (Fusco et al., 2019; Maru and Hippo, 2019). Comparing with broadly used cancer cell lines, PDOs more closely represent the heterogeneity and histomorphology of patient tumors (Kizilkurtlu et al., 2018; Fusco et al., 2019; Maru and Hippo, 2019). Drug screening based on the PDOs has recently been used as a practical strategy for drug discovery of cancer therapies (Li et al., 2016; Kondo and Inoue, 2019; Li and Izpisua Belmonte, 2019; Rowe and Daley, 2019).

In this study, to discover agents that can overcome Cisplatin resistance, we performed a drug screen with a natural product library on PDOs from Cisplatin resistant lung cancer patients (PDOs^CR^) and found Solamargine was one of the top compounds that the PDOs^CR^ were sensitive to. Solamargine is a steroidal alkaloid glycoside, naturally produced in plants of theSolanaceae family, and can also be extracted from a traditional Chinese medicinal herb, Solanum nigrum L. Solamargine has been reported to have various anti-cancer effects, such as inhibition of cell proliferation, migration, invasion (Kuo et al., 2000; Sani et al., 2015; Fekry et al., 2018), and induction of apoptosis ((Kuo et al., 2000), (Xie et al., 2015; Zhang et al., 2018)). Many studies have shown that Solamargine is involved in the regulation of signaling pathways in a variety of cancers, such as suppression of prostaglandin E2 pathway and inhibition of Stat3 phosphorylation (Zhou et al., 2014; Chen et al., 2015; Xiang et al., 2016; Fu et al., 2019). However, most of the studies only used long-term passaged cancer cell lines to test the function of Solamargine *in vitro*. So far, the response of PDOs generated from primary tumors of lung cancer patients to Solamargine is rarely reported, and the mechanism of the sensitivity of the PDOs^CR^ to Solamargine is still unknown.

The sonic hedgehog (SHH) signaling pathway has been intensively studied in cancers. The SHH pathway has an essential role in the control of cell destination in embryonic tissues and is critical in cell differentiation during tissue development (Robbins et al., 2012; Brechbiel et al., 2014). Although the SHH pathway is inactivated in adult tissues under normal circumstances, dysregulation of the SHH signaling pathway closely links with tumor development and progression (Pasca di Magliano and Hebrok, 2003; Lauth and Toftgard, 2007; Lospinoso Severini et al., 2019). Accumulating evidence indicates that the SHH pathway could be responsible for drug resistance such as platinum-based chemotherapy (including Cisplatin) resistance in non-small cell lung cancer (Barr et al., 2013; Giroux Leprieur et al., 2016; Ishiwata et al., 2018; Dwivedi et al., 2019). Discovering novel agents to sensitize Cisplatin is valuable for drug discovery and clinical practice (Zhang and Hung, 1996). In this study, we aimed to explore the underlying mechanism of the anti-cancer function of Solamargine and determine whether it is suitable for therapeutic sensitizer in Cisplatin-resistant lung cancer.

## Materials and methods

### Tissue processing and organoid culture

Lung cancer organoids were derived from surgical samples or transbronchial biopsies of lung cancer patients at Beijing Chest Hospital, Capital Medical University, Beijing, China. The study was approved by the Ethical Committee of Beijing Chest Hospital, Capital Medical University (Trial No. 48, 2018). Patients participating in this study were all consented. Fresh tumor tissues were washed with cold PBS, cut into small pieces, washed with Advanced DMEM/F12 (Thermo Fisher Scientific, Waltham, MA, United States of America; containing 1× Glutamax, 10 mM HEPES and antibiotics) and digested with collagenase (Sigma-Aldrich, St Louis, MO, United States of America; Cat. No. C9407, 2 mg/ml) for 1–2 h at 37°C. Dissociated cells were washed twice with fresh medium (containing 2% fetal calf serum, FCS) and pelleted by centrifugation (400 g, 4 min), then seeded into 10% (v/v) growth factor-reduced Matrigel (Corning Inc., Corning, NY, United States of America) supplied with Advanced DMEM/F12 at 37°C for 30 min in 24-well low binding plate. After Matrigel solidified, each well was filled with 500 μl complete human organoid medium (HOM), which was Advanced DMEM/F12 supplemented with additives as described by Lampis et al. (Lampis et al., 2017) and Loredana et al. (Puca et al., 2018). The medium was changed every 3 days. When the size of the organoid reached up to 200–500 μm in diameter (in about 1 week after plating), organoids were dissociated and passaged weekly using TrypLE Express (Gibco, Grand Island, NY, United States of America). The PDOs (2 × 10^6^ cells/tube, passage 3) were stored in the Recovery Cell Culture Freezing Medium (Gibco) at −80°C before the drug screening.

### Histology and imaging

Tissue and organoids were fixed in 4% paraformaldehyde followed by dehydration, paraffin embedding, sectioning and standard H&E staining. H&E staining images were taken with Olympus DP73. A bright-light microscope (LEICA, Wetzlar, Germany) was used for organoids bright field imaging.

### Compound screening

A collection of 1,121 natural products were obtained from MedChemExpress (Shanghai, China). The natural product library was reformatted into 96-well plates with a concentration of 3.3 μM for automated robotic screening. The cells were also treated with equal volume (0.1%) of DMSO as negative control and 1 μM Staurosporine (MCE, shanghai, China) as a positive control. Plate-to-plate normalization and assay quality control were calculated according to the controls. Cell viability assay was performed using a commercially available luminescence detection reagent (CellTiter-Glo #G9683, Promega, Madison, WI). Briefly, Pt-001 organoids were processed as described earlier and plated in a 96-well low binding assay plate with 6,000 cells per well in 50 μl 10% growth factor reduced Matrigel. An additional 40 μl culture medium without Matrigel was added above. Organoids were maintained in the medium described earlier for 48 h and 10 μl culture medium comprised of 33 μM natural products were added to each well to receive a final concentration of 3.3 μM. After 5 days of treatment, 50 μLCellTiter-Glo was added into each well and bioluminescence was measured by FLUOstar Omega (BMG). Assay quality and robustness were evaluated with signal window (SW) and Z factor (Zhang et al., 1999). Triplicate wells treated with Staurosporine (1 uM, service as positive control) and vehicle solution (DMSO) were employed as bottom wells and top wells, respectively. The assay showed the signal windows (SW) were much larger than 10 and the Z factor values were between 0.5 and 1, which indicates the assay was qualified for high-throughput screening (Supplementary Figures S1A,B).

### Cell lines and cell culture

Human lung cancer cell lines, Calu-1, Calu-3, NCI-H1299, NCI-H838, LTEP-S, NCI-H1650, NCI-H1975, NCI-H226, NCI-H460, NCI-H520, NCI-H820, PC-9 and SW1573 were purchased from the American Type Culture Collection (ATCC; Manassas, VA, United States of America). Calu-1 cells were cultured in McCoy’s 5a Medium (Gibco), SK-MES-1 and SW1573 cells were cultured in DMEM medium (Gibco) supplemented with 10% FCS, and the other cancer cell lines were maintained in RPMI-1640/1641/1,642 medium (Gibco) supplemented with 10% FCS (Gibco) and 1% penicillin-streptavidin (Gibco). All cells were cultured at 37°C in 5% CO_2_.

### Cell viability assay and foci assay

NCI-H460 (0.75 × 10^3^ cells/well) and NCI-H1299 (0.75 × 10^3^ cells/well) cell lines were seeded into 96-well plates and treated with vehicle or Solamargine for 1, 3, and 5 days. After incubation, cells were examined as described above for cell viability assay. As for the foci assay, NCI-H460 (2 × 10^3^ cells/well) and NCI-H1299 (2 × 10^3^ cells/well) were seeded into 6-well plates and treated with Solamargine for 3 days, then colonies were fixed and stained with crystal violet solution.

### Analysis of cell cycle arrest and apoptosis

Cell cycle and apoptosis were detected as previously described (Shi et al., 2018). Cells were cultured and treated with DMSO and Solamargine (2.5 μM or 7.5 μM) in both NCI-H460 and NCI-H1299 for 48 h followed by single staining with PI (Betotime) for cell cycle analysis, and dual staining with PI and Annexin V-FITC (Betotime) for apoptosis analysis with NovoCyte3130 flow cytometer.

### Western blot analysis

Western blot analysis of whole-cell protein lysates was performed using primary antibodies (1:1,000 dilution) against PARP (#9542; CST), Cleaved Caspase-3 (#9661; CST), CylclinD1 (#2978; CST), p21 (#2947; CST), β-actin (#60008-1-Ig, Proteintech).

### RNA-seq analysis

NCI-H460 and NCI-H1299 cells were incubated with DMSO or Solamargine (2.5 μM or 7.5 μM) for 48 h. Total RNA was extracted using TranZol™ UP Plus RNA Kit. RNA was sent to BGI (Beijing, China) for sequencing and analysis. Briefly, total RNA was fragmented, and mRNA was enriched using oligo (dT) magnetic beads, followed by cDNA synthesis. Double-stranded cDNA was purified and enriched by PCR amplification, after which the library products were sequenced using BGIseq-500. The heatmap of differentially expressed genes (DEGs) (log_2_FC ≥ 1, *p* ≤ 0.001) and KEGG analysis (log_2_FC ≥ 1, *p* ≤ 0.05) in NSCLC cell lines were performed by the BGI, using the Dr. TOM approach, a customized data mining system from BGI. Altered (upregulated or downregulated) expression of genes was expressed as log_2_FC, which represents log-transformed fold change ( $\log_{2}FC=\log_{2}\left[B\right]-\log_{2}\left[A\right]$ , while A and B represent values of gene expression for different treatment conditions).

### Gli responsive element reporter gene assay

GRE reporter gene assay was conducted in NIH3T3-GRE-Luc cells according to the methods described before (Lu et al., 2014; Lu et al., 2017). Briefly, NIH3T3 cells (CRL-1658, ATCC) were transfected with GRE reporter plasmids and selected with 400 ng/ml hygromycin for 3 weeks. Stable clones were isolated for assay development. The cells were re-suspended in assay medium (0.5% serum-containing DMEM) and seeded in 96-well plates. Testing compounds accompanied with 50 nM SAG (ABIN629346) were added to the assay medium. Cell plates were incubated at 37 °C for additional 48 h. Then 40 ml/well of luciferase media (Bright-Glo, Promega) was added. The plate was incubated at room temperature for 5 min under gentle shaking. Luminescence signal was measured with a plate reader (FLUOstar Omega, BMG). The IC_50_ of compounds was calculated based on the inhibition of luminescence signaling.

### BODIPY-cyclopamine binding assay

A fluorescence-based BODIPY-cyclopamine binding assay was conducted to evaluate the binding of Smo agonists/antagonists according to methods described before (Yang et al., 2015; Lu et al., 2017). Briefly, U2OS-Smo cells, stably overexpressing human Smo protein, were maintained in DMEM with 4 mM L-Gln, 1.5 g/L sodium bicarbonate and 4.5 g/L glucose, supplemented with 100 ng/ml puromycin and 10% FBS. BODIPY-cyclopamine was purchased from Toronto Research Chemicals and dissolved in methanol. U2OS-Smo cells were cultured for 48 h in a 96-well-plate and fixed with 4% paraformaldehyde (PFA). After staining with DAPI (5 mg/ml), cells were incubated for 2 h at room temperature in PBS containing 100 nM BODIPY-cyclopamine and testing compounds for competitive binding. After incubation, the cells were washed 3 times with PBST (PBS buffer supplied with 0.05% Tween-20). The fluorescence images were captured and analyzed by Arrayscan. Vismodegib was used as a reference compound to normalize the data. IC_50_ values were calculated with GraphPad Prism.

### Dual drug combination assay

NCI-H1299 and NCI-H460 cells were plated in 96-well plates and treated with various concentrations of Cisplatin or/and Solamargine, either alone or in combination for 72 h. Cell viability was determined as described above. Surface plot and heatmap of the Excess over the Highest Single Agent (EOHSA) were used to represent the difference in cell growth inhibition between the combination treatment and the most effective single compound at the corresponding concentration (Borisy et al., 2003; Simmons et al., 2014). The dose-effective curve of Cisplatin/Solamargine with the existence of Solamargine/Cisplatin was generated with GraphPad Prism. The combination index (CI) was calculated as described before by Chou Talay. CIs of <1, = 1, and >1 indicate synergism, additive effect, and antagonism, respectively (Chou, 2010).

### Statistical analysis

Data statistical analysis was performed using Prism 6.4. The Solamargine IC_50_ values were analyzed using nonlinear regression (curve fit). The 95% confidence interval was calculated. The difference in IC_50_ values between organoids and lung cancer cell lines was analyzed using the non-parameter Mann-Whitney *U* test. Cell cycle and apoptosis data were analyzed withone-way ANOVA. *p* < 0.05 was considered statistically significant.

## Results

### Derivation of the PDOs^CR^ models

**FIGURE 1:**
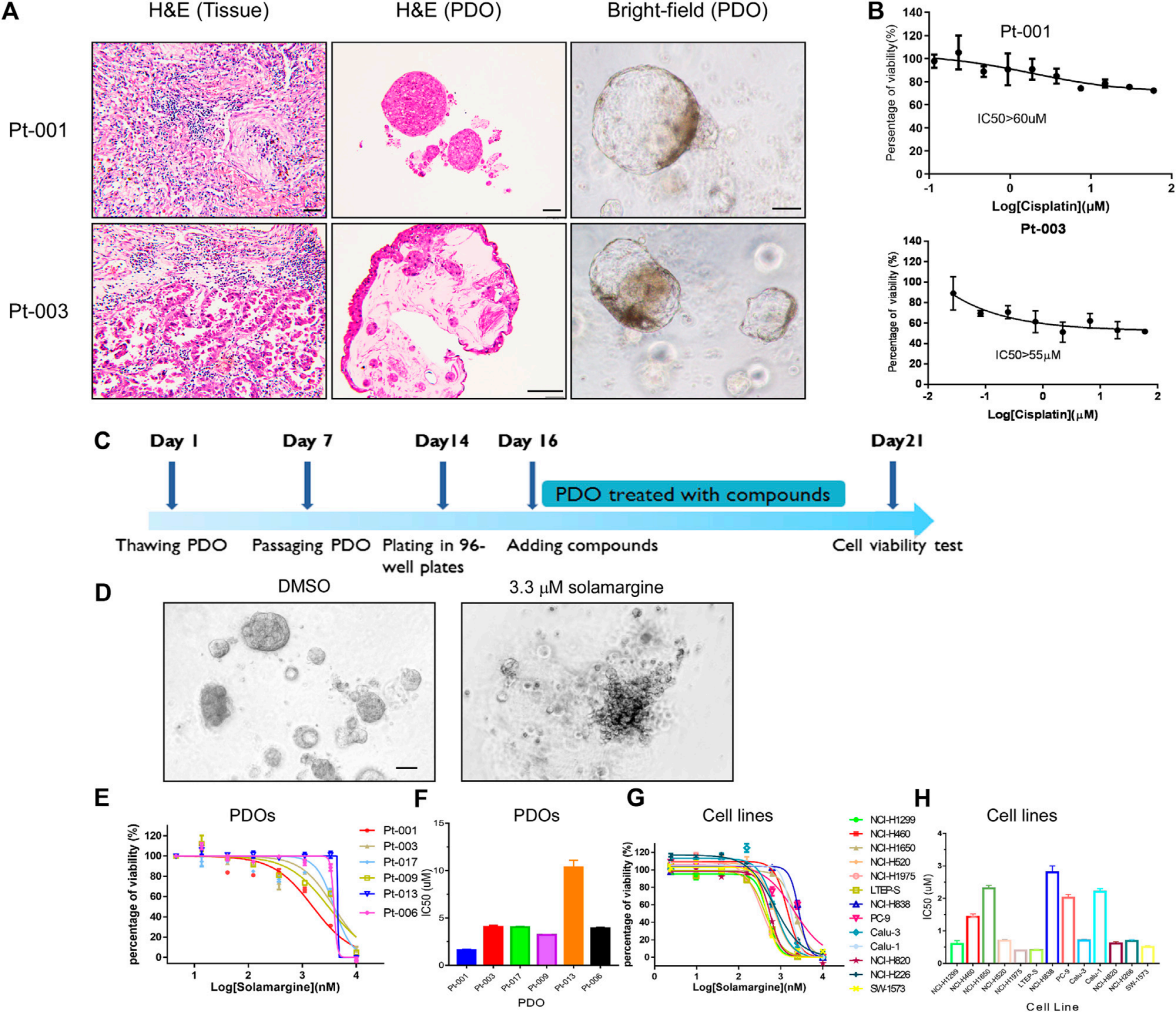
Solamargine is a potential anti-tumor agent in Cisplatin-resistant cells. **(A)** Representative histologyof primary lung cancer tissue and their tumor-derived organoids. Left and middle images are the morphology of Pt-001 and Pt-003 primary tumor tissues and their derived organoids by H&E staining. Right images are the Pt-001 and Pt-003-derived organoids in bright-field. Original magnification ×200, scale bar 200 μm. **(B)** The dose-response curve of Cisplatin to Pt-001andPt-003 organoids. N = 3. **(C)** The treatment scheme of drug screening using lung cancer patients derived organoids. **(D)** Morphological change of lung cancer patients derived organoids when treated with DMSO or 3.3 μM Solamargine for 5 days. **(E–H)** The dose-response curve of Solamargine was evaluated in thirteen lung cancer cell lines and six lung cancer patient tumor-derived organoids, respectively. Cell lines and organoids were treated for 5 days.

To perform anti-tumor drug screening using clinically relevant models for lung cancer, tumor organoids were derived from fresh tumor tissues of six lung cancer patients, obtained from surgery. The detailed patients’ information was shown in Table 1 and Supplementary Table S1. Tumor organoids were cultured in Matrigel supplemented with culture medium. These PDOs resembled their parental primary tumors in histopathological features and therapeutic resistance. In representative cases, the histology of the primary tumor from patient Pt-001 showed partially differentiated adenocarcinoma and the organoids derived from this tumor exhibited a solid growth pattern (Figure 1A). Pt-001 was derived from a 62-year-old patient, who received four cycles of pemetrexed plus Cisplatin treatment and quickly progressed. Consistently, Pt-001 showed Cisplatin resistance in cell viability assay with IC50 above 60 μM and maximal inhibition rate of 28% (Figure 1B). Organoids derived from Pt-003 demonstrated cystic structures, which recapitulated the glandular structures of the primary tumor (Figure 1A). Further, Pt-003 showed Cisplatin resistance as well (Figure 1B). The other four PDOs were not Cisplatin resistant. Pathological and pharmacological characterization demonstrated the establishment of clinically relevant models for compound screening assay development.

**TABLE 1:** Characteristics and clinical history of all patients included in the PDOs.

PDO number	Gender	Pathological pattern	Age	Tissue type	Lymphatic metastasis	T	N	M	Stage
Pt-001	male	Adenocarcinoma	62	surgery sample	10/24	4	2	1	IV
Pt-003	male	Adenocarcinoma	60	surgery sample	0/2	1	0	1	IV
Pt-006	female	adenocarcinoma	72	surgery sample	0/39	1b	0	0	IA
Pt-009	male	adenocarcinoma	50	surgery sample	0/15	2	0	0	IIA
Pt-013	female	adenocarcinoma	50	surgery sample	0/8	1	0	0	IA
Pt-017	male	squamous carcinoma	37	surgery sample	3/32	3	2	0	IIIB

### Solamargine inhibited cell growth in PDOs and lung cancer cell lines

Next, we performed cell viability assay using PDOs^CR^to find novel anti-tumor natural products (Supplementary Figures S1A,B). A single concentration of 3.3 μM was used as primary screening with a library of 1,121 natural products. In the screening, we defined the effective threshold as ≥ 50% decrease in cell viability in comparison to the vehicle control. One of the effective anti-tumor natural products, Solamargine (Supplementary Figure S2A), an alkaloid natural product, displayed >90% of cell viability inhibition in the primary screening (Figures 1C,D), so we further validated the anti-tumor effects of Solamargine on additional PDOs and multiple lung cancer cell lines. PDO from Pt-001 together with additional five lung cancer organoids derived from different patients were treated with Solamargine with serial dilution. Among the six PDOs, Pt-013 was resistant to Solamargine, with IC_50_ above 10 μM. Pt-001, Pt-003, Pt-006, Pt-009, and Pt-017 were all sensitive to Solamargine with IC_50_ of 1.6, 4.1, 3.9, 3.2, and 4.0 μM, respectively (Figures 1E,F).

To further assess the anti-tumor capability of Solamargine, we tested 13 lung cancer cell lines with Solamargine. The IC_50_ of Solamargine in NCI-H1299, NCI-H460, NCI-H1650, NCI-H520, NCI-H1975, LTEP-S, NCI-H838, PC-9, Calu-3, Calu-1, NCI-H820, NCI-H226, and SW-1573 cells were about 0.6, 1.4, 2.3, 0.7, 0.4, 0.4, 2.8, 2.0, 0.7, 2.2, 0.6, 0.7 and 0.5 μM, respectively (Figures 1G,H).

**FIGURE 2:**
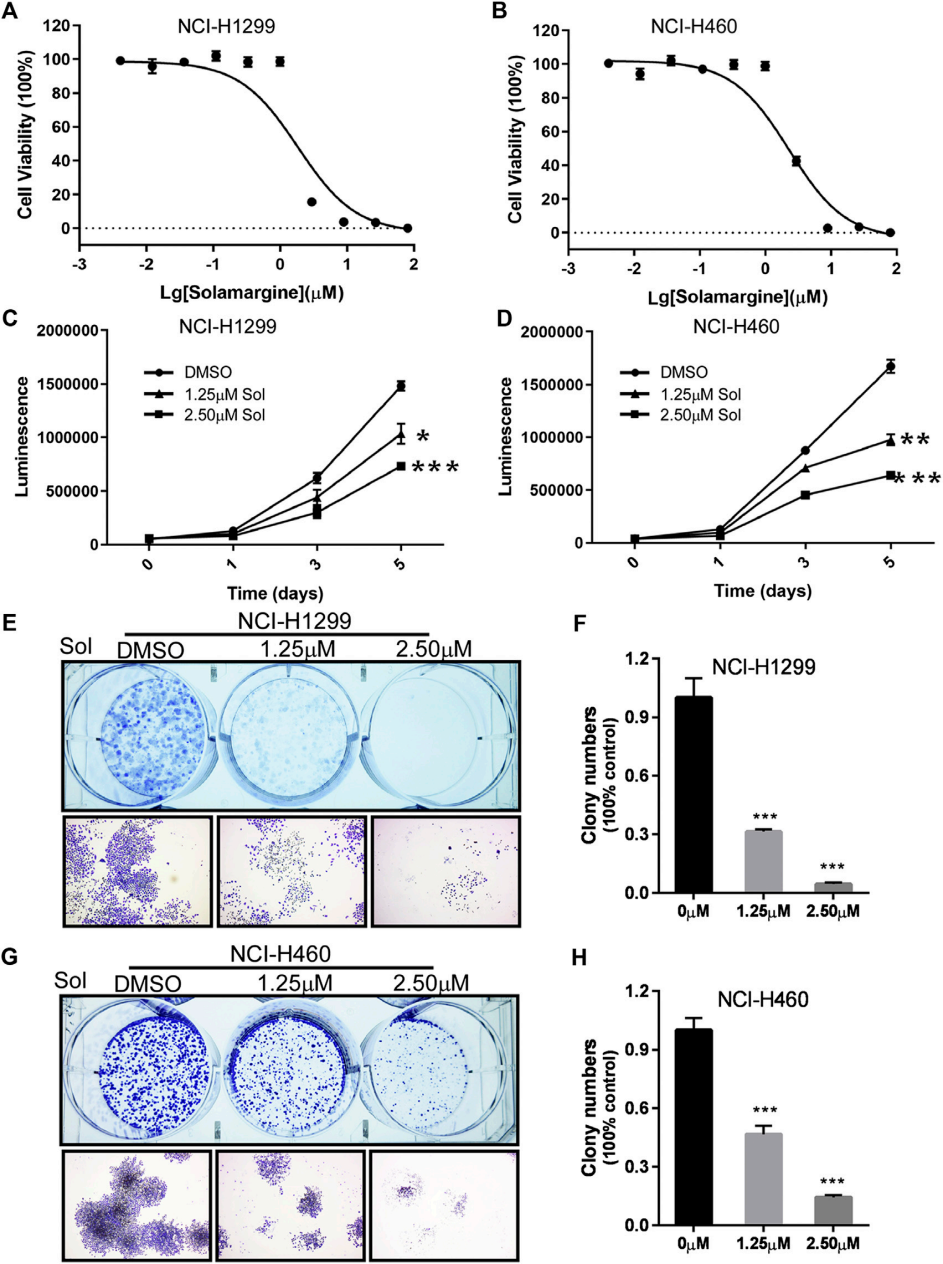
Solamargine significantly inhibited cell growth in NCI-H1299 and NCI-H460 cells. **(A,B)** The dose-response curves and IC_50_ values of Solamargine in NCI-H1299 and NCI-H460, respectively. N = 3. **(C,D)** Cell proliferation suppression in NCI-H1299 and NCI-H460 with the treatment of Solamargine at the concentration of 1.25 and 2.5 μM for 5 days. N = 3. **(E–H)** Solamargine inhibited the colony formation in NCI-H1299 and NCI-H460 cells at the concentration of 1.25 and 2.50 μM for 7 days. Cells were stained with crystal violet solution at the endpoint. N = 3. One-way ANOVA was used for statistical tests. *p < 0.05.

To further investigate the effect of Solamargine on Cisplatin-resistant models, we selected Cisplatin-resistant cell lines, NCI-H1299 and NCI-H460 (Barr et al., 2013; Xu et al., 2014) respectively. Cell proliferation was inhibited for both NCI-H1299 and NCI-H460 cells under treatment with 1.25 or 2.5 μM Solamargine compared with vehicle control in a dose-dependent and time-dependent manner (Figures 2A–D). Consistently, Solamargine decreased colony formation capacity in both cell lines in a dose-dependent manner (Figures 2E–H). The results demonstrated an anti-proliferation function of Solamargine in Cisplatin-resistant lung cancer cell lines.

### Solamargine induced cell cycle arrest and apoptosis in cisplatin-resistant lung cancer cell lines

**FIGURE 3:**
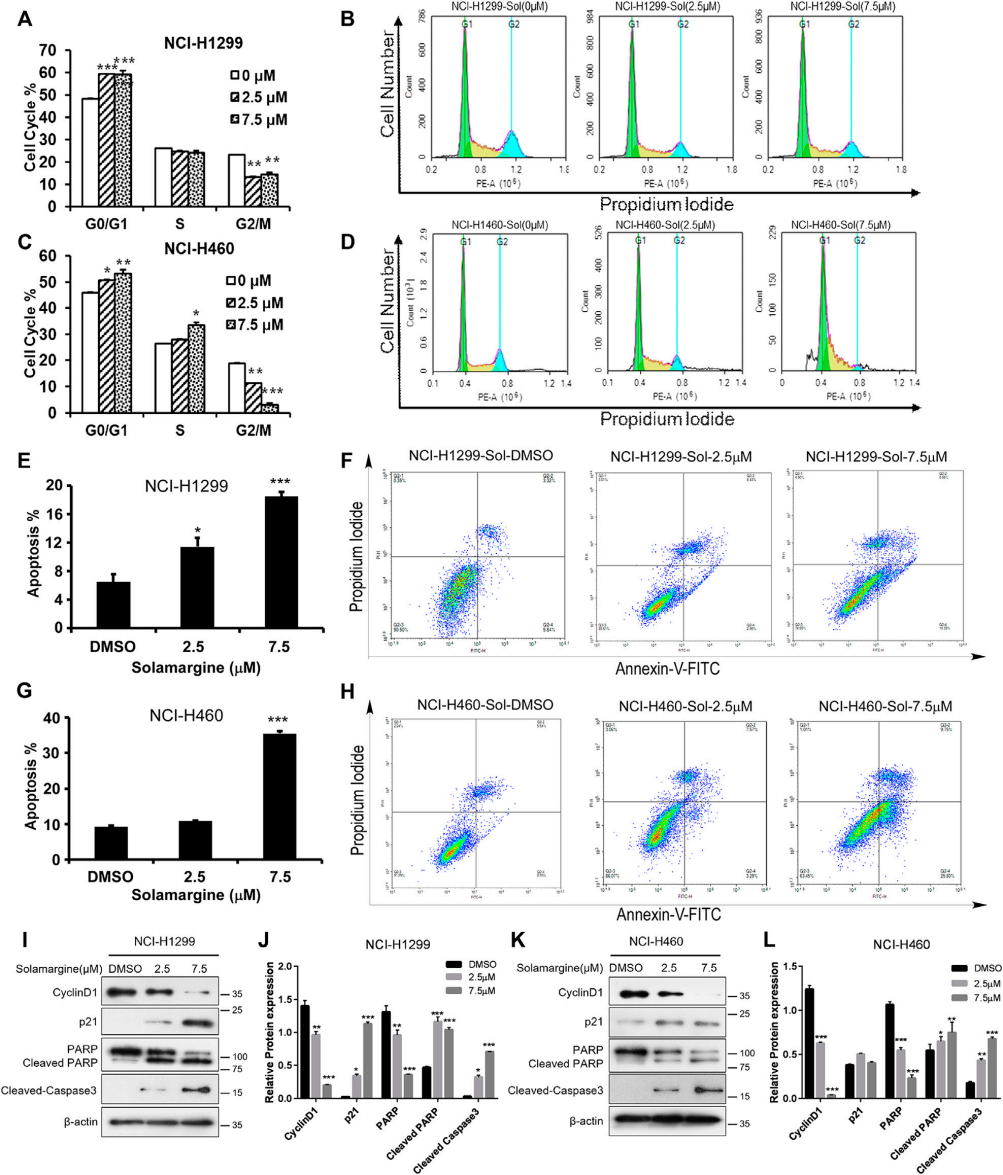
Solamargine induced G1/G0 arrest and apoptosis in NCI-H1299 and NCI-H460 cells. **(A–D)** Flow cytometry analysis of cell cycle by PI staining in NCI-H1299 and NCI-H460 cells with treatment of DMSO or Solamargine for 48 h. N = 3. **(E–H)** Flow cytometry analysis of apoptosis analysis by *p*I/Annexin V-FITC staining in both NCI-H1299 and NCI-H460 cells with treatmentof DMSO or Solamargine for 48h.N = 3. **(I–L)** Cell cycle and apoptosis markers (cyclinD1, p21, PARP, cleaved-PARP and cleaved-caspase 3) analysis by western blot for NCI-H1299 and NCI-H460 cells with treatment of DMSO or Solamargine for 48 h β-Actin was used as a loading control. N = 3. One-way ANOVA was used for statistical tests.*p < 0.05, **p < 0.01, ***p < 0.001.

To understand the mechanism of anti-proliferation activity of Solamargine, cell cycle and apoptosis analysis were conducted in NCI-H1299 and NCI-H460 cells. The cell cycle was analyzed by DNA content measurementwith flow cytometry. Increased percentage of cells in G0/G1 phase and decreased percentage of cells in G2/M phase was observed for NCI-H1299 with the treatment of Solamargine (Figures 3A,B), while in NCI-H460 cells, a significant decrease of G2/M phase and accumulation of S phase implied DNA replication dysregulation (Figures 3C,D). We also investigated whether Solamargine could induce apoptosis in lung cancer cells. With flow cytometry analysis of Annexin V and propidium iodide in Solamargine-treated NCI-H1299 and NCI-H460 cells, we found both low (2.5 μM) and high (7.5 μM) doses of Solamargine significantly increased early and late apoptosis/necrosis in NCI-H1299. NCI-H460 cells were more resistant to Solamargine-induced apoptosis and a significant increase of apoptotic cells was only observed in high dose treatment (Figures 3E–H). We further confirmed the anti-proliferative and pro-apoptotic effects of Solamargine by measuring critical proteins involved in these processes. By western blot, 48-h treatment of Solamargine downregulated CyclinD1 and upregulated P21, cleaved PARP, and Caspase3 (Figures 3I–L). Together, our data indicated that the drug sensitivity caused by Solamargine to NCI-H1299 and NCI-H460 was related to decreasing proliferation and inducing apoptosis.

### Solamargine altered genome-wide gene expression in cisplatin-resistant lungcancer cell lines

To further reveal the underlying molecular mechanism, RNA-seq analysis was performed to profile transcriptomes in both NCI-H1299 and NCI-H460 with the treatment of lower dose (2.5 μM) and higher dose (7.5 μM) of Solamargine. We identified 257 differentially expressed genes (DEGs) (log_2_FC ≥ 1, *p* ≤ 0.001) in NCI-H1299with 2.5 μM Solamargine treatment and 1,013 DEGs (log_2_FC ≥ 1, *p* ≤ 0.001) with 7.5μMSolamargine (Supplementary Figures S2A,B). 115 interactions of DEGs (log_2_FC ≥ 1, *p* ≤ 0.001) were identified in NCI-H1299 with both treatments (Supplementary Figure S2C). The number of DEGs identified in NCI-H460 was less than that in NCI-H1299, which might be caused bythe sensitivity difference between the 2 cell lines (Supplementary Figures 2D–F). KEGG analysis of the DEGs (log_2_FC ≥ 1, *p* ≤ 0.05) showedthat pathways in cancerwerethe most significantly enriched pathways in the treated cells. (Supplementary Figure S2G).

### Solamargine inhibited hedgehog signaling pathway by targeting SMO

**FIGURE 4:**
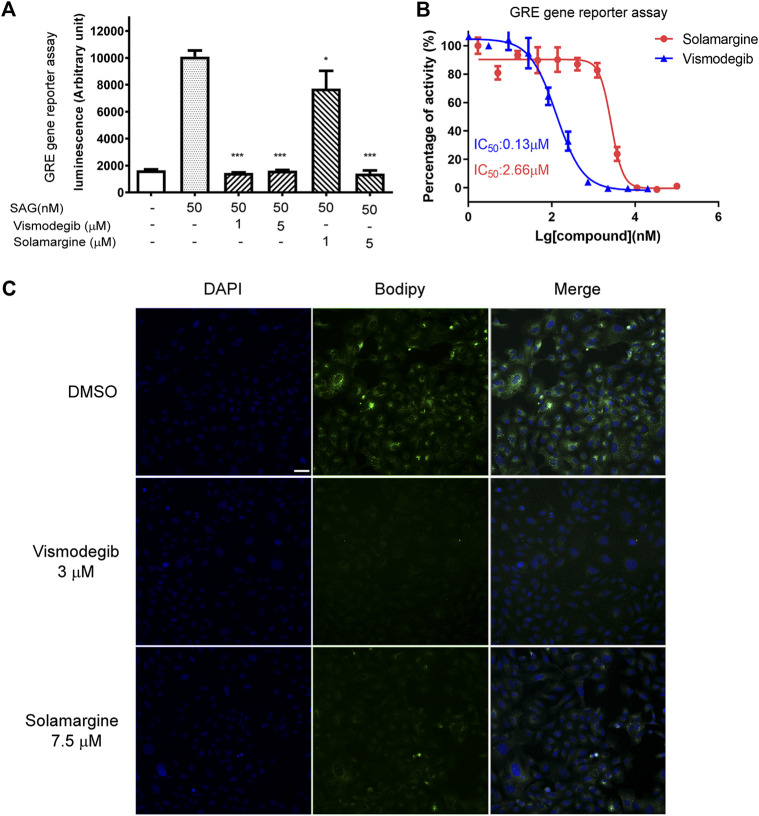
Solamargine inhibited the Hedgehog signaling pathway by direct binding to smoothened protein. **(A)** NIH3T3 cells stably transfected with GRE2-pGL4.26 were plating 15,000 cell/well into 96-well-plates in the assay medium and treated with DMSO (0.1%), vismodegib or Solamargine (1 and 5 μM). Gli responsive element (GRE) reporter gene assay was used to measure the SHH pathway activity. N = 3. **(B)** The dose-response curve of Solamargine and vismodegib in NIH3T3 cells which were stably transfected with GRE2-pGL4.26. **(C)** 2 × 10^4^U2OS cells were seeded into each well, then fix the cells DAPI: nuclear staining (the left lane) the next day, or add BODIPY-Cyclopamine (100nM, the middle lane) and Solamargine (7.5 μM) or vismodegib (3 μM) co-incubated for 2 h at RT, then measure the fluorescence signal intensity using a confocal microscope. Scale bar 200 μm.One-way ANOVA was used for statistical tests.*p < 0.05.

Based on our KEGG analysis that pathways in cancer were the most significantly enriched pathways in Solamarg treated cells and the Hedgehog pathway is associated with cancer and several hedgehog signaling pathway inhibitors such as vismodegib and sonidegib have been developed for cancer treatment (Ooft et al., 2019). Furthermore, *in silico* molecular docking data predicted that Solamargine, Solasonine and Tylophorine were good candidates to target CSCs by modulating the hedgehog pathway through binding to the sonic hedgehog, smoothened and GLI proteins (Mayank and Jaitak, 2016), which promptedus to further analyze the effect of Solamargine on hedgehog signaling pathway. GREreporter gene assay was performed to study the regulation of GLI by drugs. GLI-reporting plasmid, GRE-pGL4.26 plasmid was generated by inserting GLI-responsive element into the promoter region of pGL4.26 plasmid carrying luciferase gene. NIH-3T3 cells were transfected with the GRE-pGL4.26 plasmid and transfected clones were tested for signal intensity and quality. In general, about 10-fold luciferase activitycould be observed under treatment of smoothened agonist (SAG, Supplementary Figure S3B). The half-maximal effective concentration (EC_50_) of SAG was 73.19 nM in GRE reporter gene assay (Supplementary Figure S1C). The luminescent signal induced by SAG was significantly inhibited by the treatment of two doses of Solamargine and vismodegib (Supplementary Figure S3C) (GDC-0449), an FDA-approved SHH pathway inhibitor, as the positive control (Figure 4A). Further analysis showed Solamargine and vismodegib inhibitedhedgehog signaling in GRE gene reporter assay with IC_50_ of 2.66 and 0.13μM, respectively (Figure 4B). As GLI1 can transcriptionally regulate itself, we measured GLI1 gene expression as an alternative readout for SHH signaling activation. By RT-qPCR, SAG-induced gene expression of GLI1 was decreased by treatment of VismodegibinNIH-3T3 cells (Supplementary Figure S1D), which was consistent with the GRE reporter gene assay. To further understand the target of Solamargine in the SHH pathway, we used BODIPY-cyclopamine, a fluorescent derivative of cyclopamine that inhibited hedgehog signaling by binding directly to SMO (Lu et al., 2014; Lu et al., 2017) (Supplementary Figure S3D). As previously reported, vismodegib can directly bind to SMO and competitively remove BODIPY-cyclopamine from SMO (Lu et al., 2017), which leads to decreased fluorescent signal (Figure 4C). In U2OS cells, the fluorescent signaling of BODIPY-cyclopamine wasalso suppressed by Solamargine (Figure 4C), suggesting the inhibitory binding of Solamargine to SMO.

### Solamargine synergized with DDP in DDP-resistant cells

**FIGURE 5:**
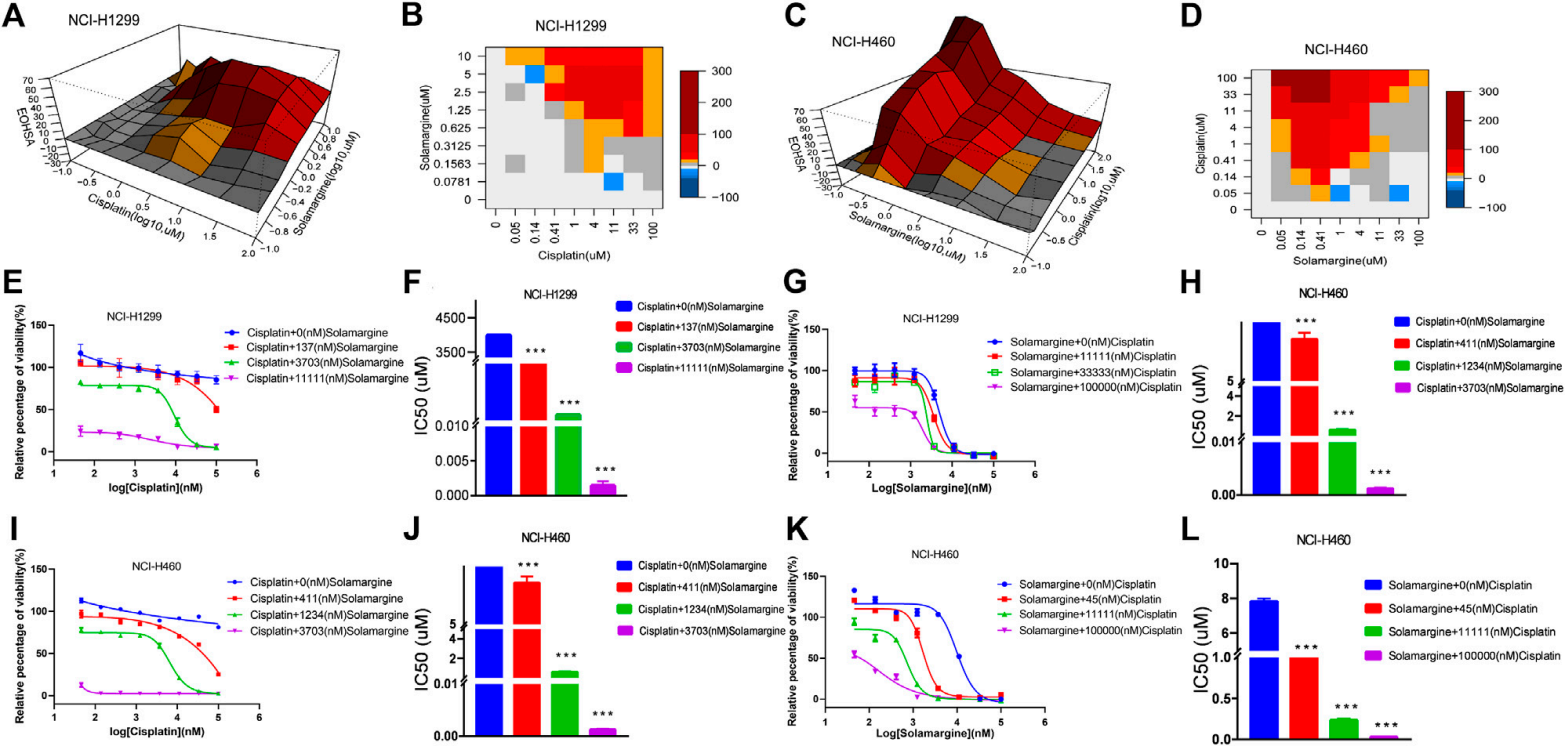
Synergy effects of Solamargine and Cisplatin on cell viability of NCI-H1299 and NCI-H460. **(A–D)** The surface plot and heatmap show the Excess over the Highest Single Agent (EOHSA) of Solamargine and Cisplatin combination in NCI-H1299 and NCI-H460 cells. **(E–H)** The dose-response curve of Solamargine/Cisplatin in the presence of Cisplatin/Solamargine in NCI-H1299 cells. **(I–L)** The dose-response curve of Solamargine/Cisplatin in the presence of Cisplatin/Solamargine in NCI-H460 cells.

Next, we investigated if Solamargine could sensitize DDP-resistant lung cancer cells to DDP. NCI-H1299 and NCI-H460 cells were treated with the serial diluted DDP with the existence of various concentrations of Solamargine for 72 h. Chou-Talalay method was employed to quantitatively evaluate the synergy effect of Solamargine with DDP. The excess over the highest single agent (EOHSA) was used to describe the difference in cell growth inhibition between the combination treatment and the most effective single compound at the corresponding concentration. Surface plots and heatmap were used to visualize the EOHSA of Solamargine and DDP (Figures 5A–D). In DDP-resistant cells, Solamargine sensitized the cells to DDP. A significant left shift of the dose-response curve was observed for DDP with the presence of Solamargine, together with a decrease of IC_50_ for DDP (Figures 5E,F,I,J). On the other hand, with the existence of DDP, the efficacy of Solamargine was also significantly increased (Figures 5G,H,K,L). Thus, these data suggested the synergistic effects of Solamargine and DDP, which was evidenced by CI value (Table 2).

**TABLE 2:** Combination index (CI) of Solamargine and Cisplatin in H1299 and H460.

Cell lines	Solamargine dose (nM)	Cisplatin dose (nM)	Mean growth inhibition (%)	Dose of solamargine alone with same inhibition (nM)	Dose of cisplatin alone with same inhibition (nM)	CI
	D1	D2	X	DX1	DX2	
**NCI-H1299**	3,704	100,000	90.21	59,680	5.13719E+14	0.06
	3,704	33,333	76.81	40,209	7.84882E+13	0.09
	3,704	11,111	23.99	8,477	47,697,255,698	0.44
	1,235	100,000	＜10	4,448	2,216,814,353	0.28
	1,235	33,333	＜10	559	115,047	2.50
	1,235	11,111	＜10	326	8,827	5.05
**NCI-H460**	3,704	100,000	97.52	53,410	9.47082E+16	0.07
	3,704	33,333	97.48	53,205	9.39261E+16	0.07
	3,704	11,111	97.24	52,095	8.95715E+16	0.07
	1,235	100,000	92.10	38,925	3.15471E+16	0.03
	1,235	33,333	87.99	33,692	1.37157E+16	0.04
	1,235	11,111	70.99	22,669	4.36952E+14	0.05

## Discussion

Cisplatin-based chemotherapies have been the standard of care for many types of cancers, including lung cancer. However, the quick emergence of resistance and systemic toxicity dampens its applications in the clinic. Therefore, identifying Cisplatin synergistic sensitizers is of great clinical significance. Accumulating evidence has indicated that aberrant activation of stem cell-related pathways contributes to Cisplatin resistance, but inhibition of stem cell pathways sensitizes cell response to Cisplatin (Tian et al., 2012; Ahmad et al., 2013; Seidl et al., 2020). In our study, we investigated the Cisplatin resistance issue by performing natural compound screening using patient-derived tumor organoids models derived from Cisplatin-resistant primary tumors. The major advantage of using patient-derived tumor organoids is the effective preservation of the main behavioral characteristics of the primary tumors, which is more clinically relevant than cancer cell lines.

Natural products are a group of chemical compounds, which are naturally found in plants, characterized by large diversity and abundance and rich resources for anti-cancer drug screening. One of the benefits of natural products is that they are commonly identified as multi-target drugs and might hit several targets at the same time, producing promising therapeutic effectsduring cancer treatment (Koeberle and Werz, 2014). Moreover, phenotype induced by multi-targeting natural products could provide valuable information about drug combinations (Zheng et al., 2014; Sanchez et al., 2019). Previous studies have reported that Solamargine assumes an anti-tumor effect on a variety of tumor types (Xie et al., 2015; Zhang et al., 2018; Fu et al., 2019); however, the role of Solamargine in Cisplatin-resistant lung cells and mechanism of action have not yet been fully investigated (Liu et al., 2004; Shiu et al., 2007; Liang et al., 2008). Herein, we successfully established Cisplatin-resistant organoids to evaluate the efficacy of natural products to suppress tumor growth. Among 1,121 natural products, Solamargine was found to be a top hit in reducing cell viability of Cisplatin-resistant PDOs. We further investigated the mechanism of Solamargine anti-tumor function and found Solamargine can induce cell cycle arrest and apoptosis in Cisplatin-resistant tumor cell lines. Pathway analysis showed Solamargine affected the SHH pathway. Previous in silico molecular docking studies indicated that Solamargine might be an inhibitor of the SHH pathway (Mayank and Jaitak, 2016; Yang et al., 2016; Butt et al., 2018).

We further investigated the function of Solamargine in regulating the SHH pathway and found that Solamargine was competitivelybound to SMO with cyclopamineindicating that SMO protein might be a target of Solamargine. Drug combination study confirmed the synergetic effect of Cisplatin and Solamargine in Cisplatin-resistant cell lines. Despite the importance of the SHH pathway in stemness and neoplasia, clinical development of vismodegib has failed in several cancer types except for basal cell carcinoma (Chen et al., 2017). However, targeting cancer stem cells by inhibiting the SHH pathwayhas been found to improve drug resistance (Giroux-Leprieur et al., 2018; Subramaniam et al., 2018). In many tumor types, the SHH signaling pathway has been reported to crosstalk with other critical molecular signaling pathways involved in cancer, such as RAS/RAF/MEK/ERK, PI3K/AKT/mTOR, EGFR, and Notch (Liu et al., 2006; Riobo et al., 2006; Morrow et al., 2009; Wall et al., 2009; Lauth, 2011; Mangelberger et al., 2012; Das et al., 2013). Numerous preclinical studies have revealed that the combination of some chemicals with SHH inhibitorsresults in improved anti-tumor efficacy and survival in animal models (Wang et al., 2012). Multi-target drugs, simultaneously blockingmultiple crucial pathways, become the trend of best-in-class drug development (Lu et al., 2012). Multi-target compounds for the hedgehog and PI3K/AKT/mTOR have been reported previously (Yang et al., 2015).

Taken together, natural productsform a huge multi-target chemical library for synergetic study and multi-target leading drug identification. By establishing clinically relevant PDO models, high-throughput screening assay and RNA sequencing, we demonstrated Solamargine could be considered as a potential therapeutic agent and sensitizer of Cisplatin for Cisplatin-resistant lung cancer.

In summary, we successfully established Cisplatin-resistant PDOs together with lung cancer cell lines to confirm the anti-cancer biology behavior of Solamargine, such as growth inhibition, G1/G0 phase arrest and apoptosis induction. Moreover, we unveiled Solamargine exhibited lung cancer suppression by targeting SMO in the hedgehog signaling pathway. Although further investigations should be done to verify whether SMO is the key target in lung cancer, our data is of great value forpromising combination therapy and would provide a possible solution for the improvement of individualized therapeutic and prognosis of lung cancer in the coming days.

## Data Availability

The datasets presented in this study can be found in online repositories. The names of the repository/repositories and accession number(s) can be found below: BioProject database, accession PRJNA814112.
