# Supplementary figures and images for: Identification of solamargine as a cisplatin sensitizer through phenotypical screening in cisplatin-resistant NSCLC organoids
Source: Front Pharmacol. 2022 Aug 10;13:802168. doi: 10.3389/fphar.2022.802168 (PMC9399411; doi:10.3389/fphar.2022.802168)

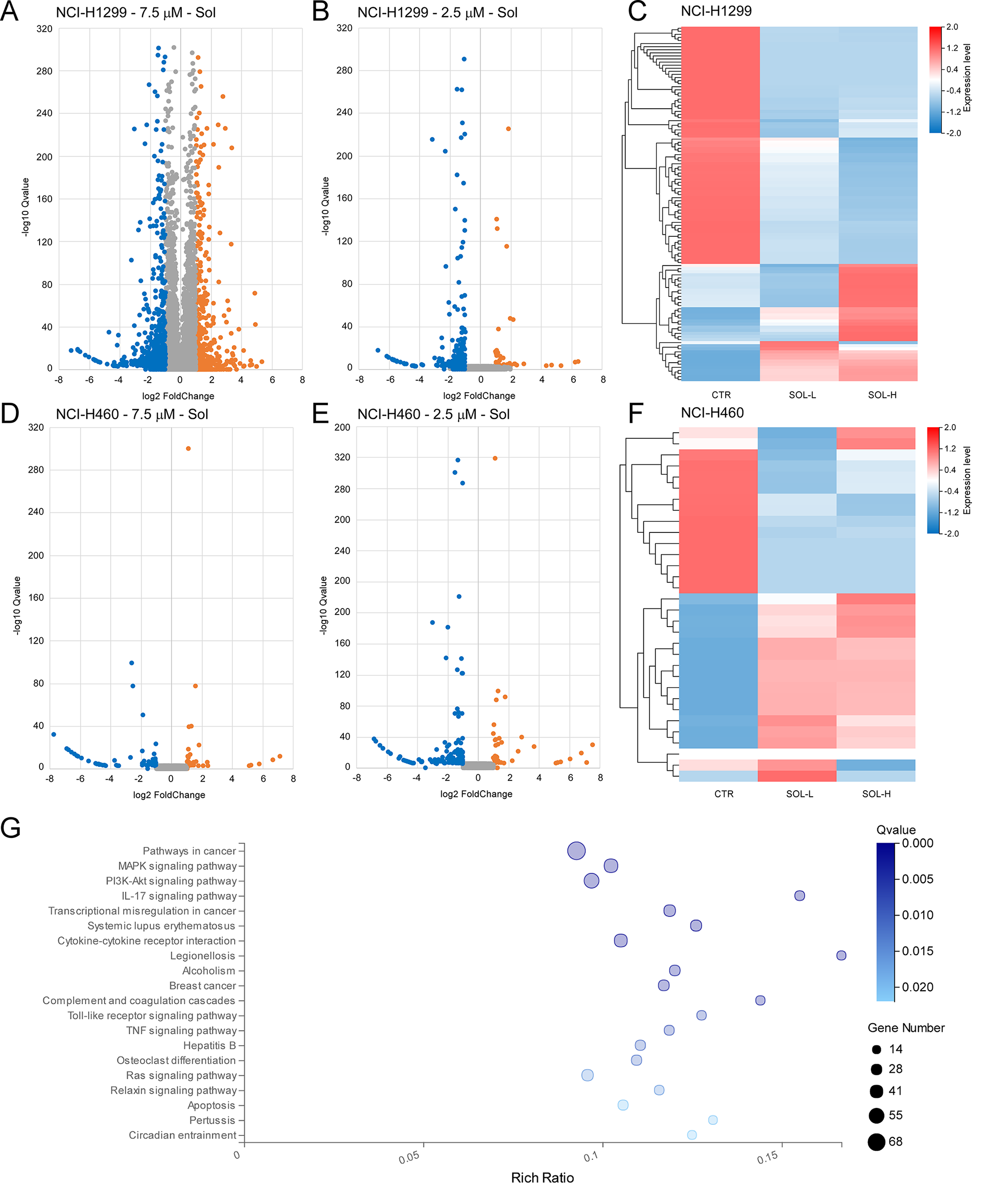

Supplement: Supplementary file 1 [file Image3.TIF]

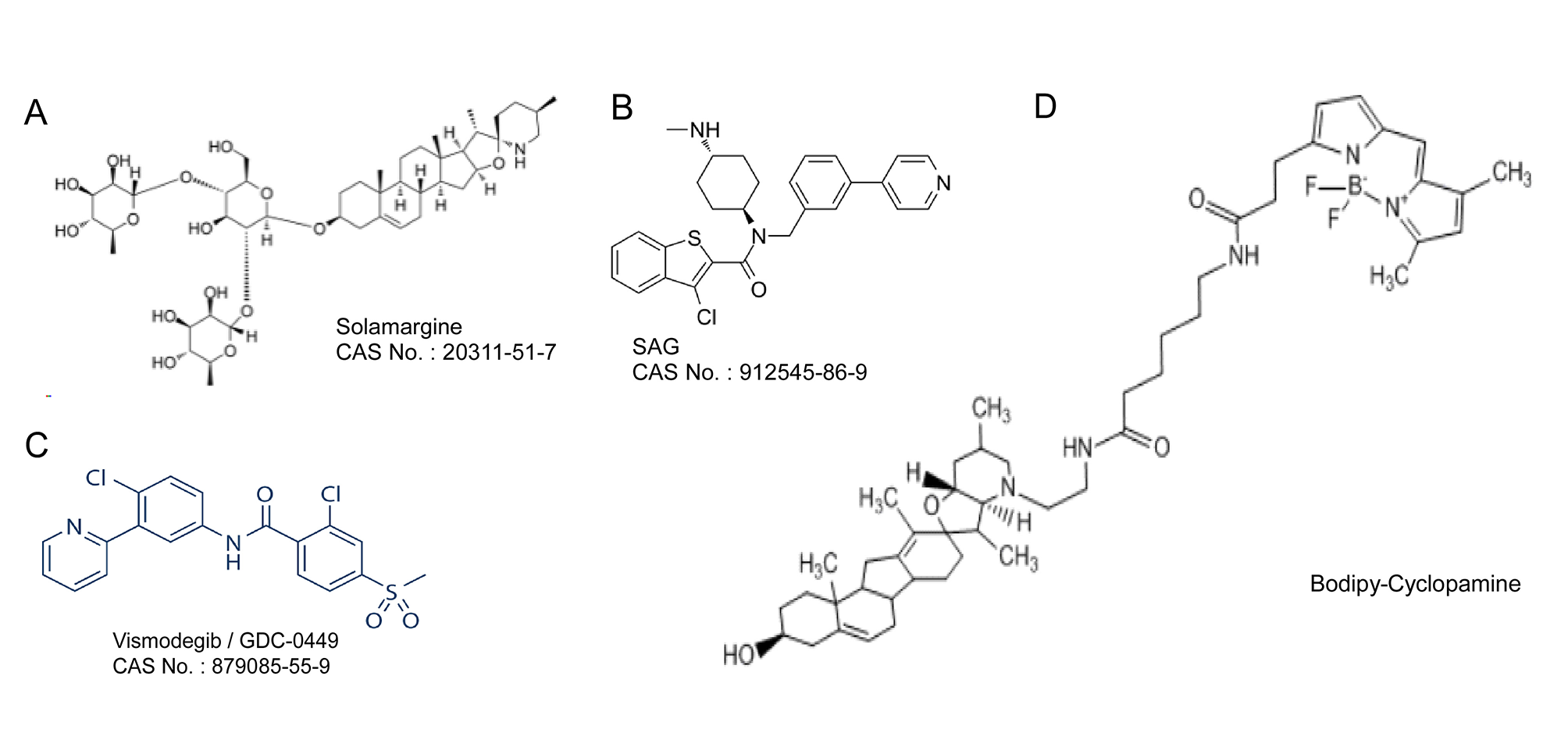

Supplement: Supplementary file 2 [file Image2.TIF]

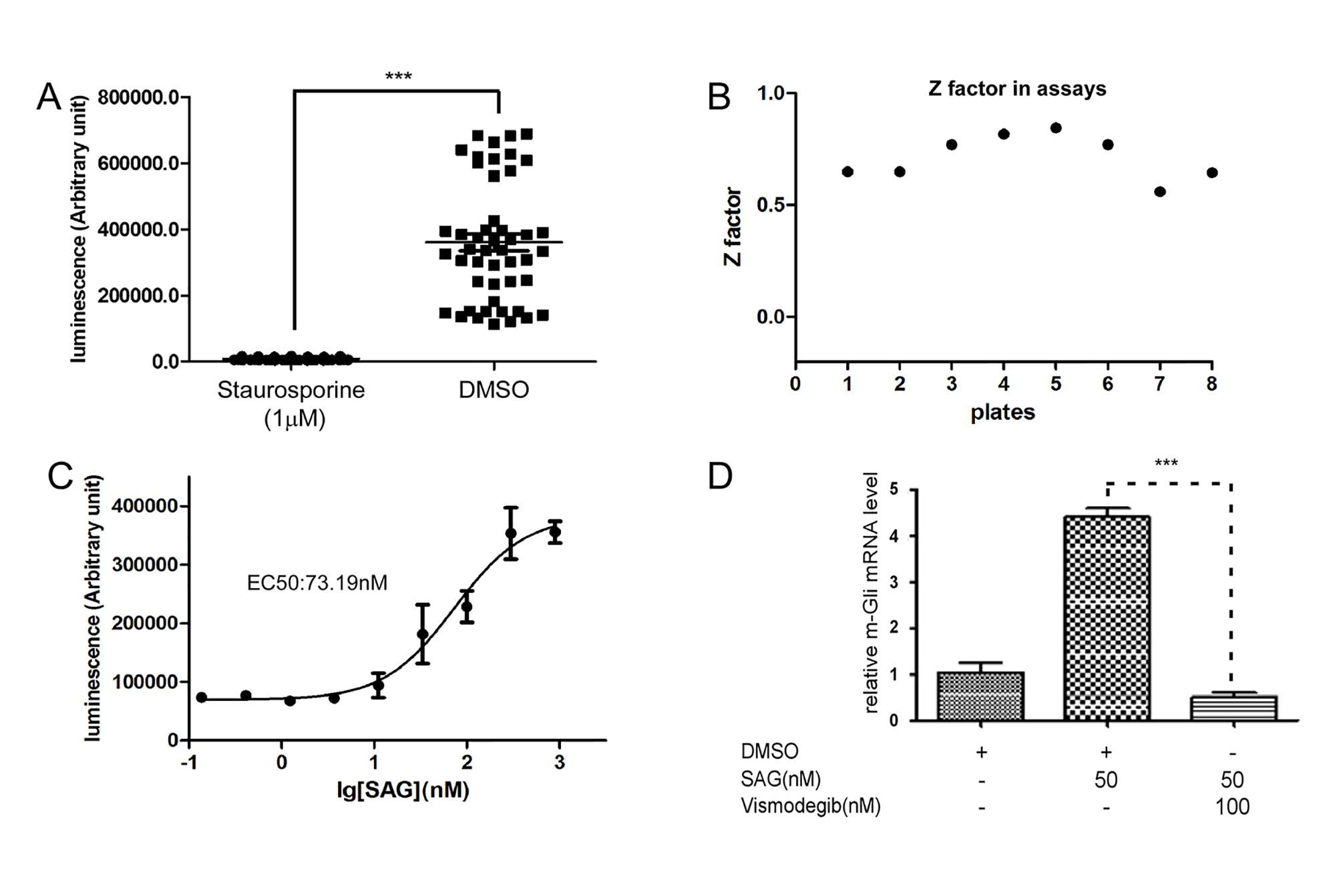

Supplement: Supplementary file 3 [file Image1.TIF]
